# The unequal cognitive consequences of social disconnection: mechanisms, modifiers and opportunities for dementia prevention

**DOI:** 10.3389/fpsyt.2026.1949322

**Published:** 2026-10-12

**Authors:** Xing Xu, Zhaoying Yu, Lei Gao, Yaping Chen, Yu Liu

**Affiliations:** 1 Department of Psychiatry, Affiliated Minkang Hospital of Ningbo University (Ningbo Psychiatric Hospital), Ningbo, China; 2 Health Science Center, Ningbo University, Ningbo, China; 3 School of Health and Care, Zhejiang Pharmaceutical University, Ningbo, China

**Keywords:** cognitive decline, dementia, loneliness, precision intervention, public health, social isolation

## Abstract

Social isolation and loneliness are increasingly recognized as important determinants of cognitive decline and dementia risk in older adults. However, the strength and nature of these associations vary substantially across populations, cognitive outcomes, and stages of ageing. This review examines the sources of such heterogeneity by distinguishing objective social isolation from subjective loneliness and exploring their different psychological, behavioural, and biological pathways. Using a systematic multi-database search and thematic synthesis, we integrated evidence from longitudinal studies, neuroimaging research, and intervention trials to identify factors that increase vulnerability and promote resilience. The review highlights how psychosocial, behavioural, neuroendocrine, and neurostructural mechanisms contribute to differences in cognitive outcomes and discusses implications for targeted and context-sensitive interventions. Remaining challenges include measurement heterogeneity, limited causal evidence, and underrepresentation of low- and middle-income countries. Future research should prioritize harmonized assessments, stratified intervention trials, and culturally adapted implementation approaches. Early identification and prevention of social disconnection may provide greater benefits than attempts to reverse established isolation.

## 1 Introduction

### 1.1 The public health significance of cognitive health in the context of global population aging

Populations worldwide are ageing at an unprecedented pace. By 2050, the number of people aged 60 years and older is projected to reach 2.1 billion, with those aged 80 years and older forming the fastest-growing segment in many regions. Cognitive impairment can reduce autonomy and quality of life and is associated with disability, institutionalization, and mortality. The global number of people living with dementia is projected to exceed 150 million by 2050, creating substantial health-system and economic burdens. Rapidly ageing societies, including China, Japan, Europe, and the United States, will carry a large share of this burden (1).

China faces a particularly pressing challenge because of the scale of population ageing and major changes in family and community life. Urbanization and internal migration have left many older adults living apart from their adult children, while traditional sources of day-to-day support have weakened. The World Health Organization recognizes social isolation and loneliness as public health concerns (2). The 2024 Lancet Commission estimated that addressing 14 modifiable factors, including depression and social isolation, could prevent or delay up to 45% of dementia cases (3). These observations support closer examination of social conditions as potentially modifiable contributors to cognitive health, while not establishing causality for any individual exposure.

### 1.2 The public health burden of social isolation and loneliness

Social isolation, the objective lack of social contacts and integration (4), and loneliness, the distress arising from a perceived gap between desired and actual relationships (5), are distinct but related constructs. In this review, social disconnection is used only as an umbrella term for these two constructs. Approximately one-quarter of older adults worldwide experience social isolation, and 20% to 34% report loneliness (6, 7). A six-continent meta-analysis reported a pooled loneliness prevalence of 26% (95% confidence interval, 23% to 30%), with substantial regional variation (8). A cross-sectional analysis of 15,874 participants aged 65 years or older in the 2018 Chinese Longitudinal Healthy Longevity Survey found a loneliness prevalence of 23.4% (9). Higher Mini-Mental State Examination (MMSE) scores were associated with lower loneliness in that study, but the MMSE is a screening instrument rather than a standalone diagnosis. At the conventional cut-off of 24, pooled community-based sensitivity was 0.85 and specificity was 0.90, with performance varying by threshold and education (10).

Loneliness and social isolation have been associated with higher risks of all-cause mortality, cardiovascular disease, stroke, depression, cognitive decline, and dementia (4, 11–14). A meta-analysis of 86 studies reported hazard ratios of 1.14 for loneliness, 1.35 for social isolation, and 1.21 for living alone in relation to mortality (13). Longitudinal meta-analyses also associate loneliness with a 26% to 31% higher risk of dementia (15). For mild cognitive impairment (MCI), the pooled association was smaller overall, but estimates were larger for new-onset MCI (16). These observational associations identify potentially modifiable risk markers, but they do not by themselves establish that reducing loneliness or isolation will prevent dementia.

### 1.3 Heterogeneity in existing research: key issues and gaps

Published estimates vary in magnitude, specificity, and sometimes direction. Important sources of heterogeneity include conflation of social isolation with loneliness, single-item versus multi-item measures, different cognitive outcomes, population composition, follow-up duration, and unequal control of confounding. Reverse causation is also plausible because prodromal cognitive decline may reduce social participation or alter perceived connection. The present review therefore treats heterogeneity as an interpretive problem rather than evidence for a single universal effect and uses it to define testable prevention questions.

### 1.4 The unique contribution of this review

High-quality meta-analyses have quantified associations between social factors and cognition (4, 15, 17), but three translational questions remain unresolved. First, which psychological, behavioural, and biological pathways may distinguish social isolation from loneliness? Second, which patterns of heterogeneity are sufficiently consistent to justify stratified intervention trials? Third, how transferable is the predominantly Western evidence base to low- and middle-income settings, particularly China? By integrating epidemiological, neuroimaging, intervention, and cross-cultural evidence, this review develops a hypothesis-generating framework for these questions.

### 1.5 Search strategy and methods

This narrative review incorporated systematic search elements to improve transparency while retaining an interpretive scope across epidemiology, neuroimaging, mechanisms, interventions, and cross-cultural evidence. A meta-analysis was not undertaken because the exposures, outcomes, measures, populations, and study designs were too heterogeneous for a single pooled estimate to address the review questions. We searched PubMed, PsycINFO, Web of Science, and Scopus database using terms for social isolation or loneliness, cognition or dementia, and older adults or ageing. Older adults were primarily defined as individuals aged 60 years or older. The screening process retained studies after quality appraisal. Findings were synthesized thematically by construct, cognitive outcome, population modifier, proposed mechanism, and intervention evidence level. Systematic reviews were appraised with AMSTAR-2. Longitudinal cohorts were appraised with the Newcastle-Ottawa Scale (NOS) because it provided a common framework for selection, comparability, and outcome assessment across heterogeneous cohorts. We acknowledge that ROBINS-E is more specific to non-randomized exposure studies and that the NOS may not capture every exposure-confounding domain. Quality judgements informed the weighting and interpretation of evidence rather than a pooled effect estimate.

## 2 Conceptual distinctions: differentiating social isolation from loneliness

### 2.1 Social isolation: definition and measurement

Social isolation is an objectively measured condition characterized by sparse networks, infrequent contact, or limited social participation (4, 13). It is not equivalent to living alone, because a person may live alone while maintaining frequent and supportive contact. The six-item Lubben Social Network Scale (LSNS-6) assesses family and friendship networks, contact frequency, and perceived support; scores of 12 or lower are commonly used to identify risk of isolation (4, 18, 19). Other studies use composite indices, including measures derived from the National Health and Aging Trends Study (NHATS), that combine household, network, contact, and participation indicators (20, 21). Because these instruments weight structural dimensions differently and use different thresholds, prevalence estimates and effect sizes are not directly interchangeable (4, 22).

### 2.2 Loneliness: definition and measurement

Loneliness is the subjective distress that arises when perceived relationships do not meet a person’s desired level of connection (5, 23, 24). The University of California, Los Angeles (UCLA) Loneliness Scale captures global perceived loneliness, whereas the De Jong Gierveld scale distinguishes emotional from social loneliness (23, 25–27). Single-item measures reduce respondent burden in large surveys but provide limited coverage of the construct and may classify respondents differently from multi-item scales (18, 28). This measurement choice matters because binary loneliness measures have produced larger dementia-risk estimates than continuous scales (15).

### 2.3 Why social isolation and loneliness cannot be used interchangeably

Social isolation and loneliness are only moderately correlated and should not be used interchangeably (29–31). Reported neuroimaging correlates also differ, with loneliness associated with thalamic and cerebellar measures and social isolation associated with hippocampal measures (19, 28, 32). These findings are not uniform. Some longitudinal cohorts found loneliness, but not isolation or social support, associated with cognitive decline or dementia (27, 33, 34), whereas another cohort found the socially isolated but not lonely group had the fastest cognitive decline (35). Differences in measurement, sample composition, follow-up, and confounder adjustment may explain part of this disagreement.

The neuroimaging evidence remains preliminary. Available studies were observational or exploratory, included samples ranging from 176 to 1,992 participants, and did not consistently report independent validation or correction for multiple comparisons (19, 28, 32). Structural differences may precede social disconnection, follow reduced engagement, or reflect prodromal cognitive decline. The reported regional associations should therefore be treated as hypothesis-generating correlates rather than established biomarkers or causal mediators (**Table 1**).

**Table 1:** Distinctions between social isolation and loneliness.

Dimension	Social isolation	Loneliness
**Definition**	Objective lack of social contacts and integration	Subjective distress from perceived discrepancy between desired and actual social relationships
**Nature**	Quantifiable state	Subjective emotional experience
**Measurement Tools**	LSNS-6 (≤12 indicates isolation), NHATS composite index, CHARLS composite index	UCLA Loneliness Scale (3/10/20-item), De Jong Gierveld Scale, single-item measures
Reported neuroimaging correlates	Hippocampal atrophy (19) (32)	Thalamic, cerebellar, and occipital grey matter reduction (28)
Proposed pathways	Cognitive stimulation deficit → Cognitive reserve depletion	HPA axis activation → Depressive symptoms → Neuroendocrine dysregulation
Candidate intervention approaches	Increase objective social participation, hearing rehabilitation	CBT, cognitive reframing, reminiscence therapy
**RCT Evidence**	**Social-contact interventions have heterogeneous effects; a single pooled cognitive effect is not established**	**CBT-based approaches reduce loneliness; effects on cognitive outcomes remain uncertain**

LSNS-6, Lubben Social Network Scale-6; NHATS, National Health and Aging Trends Study; CHARLS, China Health and Retirement Longitudinal Study; UCLA, University of California, Los Angeles; HPA, hypothalamic-pituitary-adrenal; CBT, cognitive-behavioural therapy; RCT, randomized controlled trial. Bold text identifies the row summarizing randomized controlled trial (RCT) evidence.

## 3 Evidence for heterogeneous effects

This section critically synthesizes how associations between social disconnection and cognition vary across cognitive domains, populations, and cognitive stages (**Table 2**). Rather than treating every subgroup estimate as a separate conclusion, we focus on recurring patterns, plausible sources of disagreement, and areas where evidence remains uncertain.

**Table 2:** Summary of effect sizes by domain, population subgroup, and cognitive stage.

Stratification level	Exposure	Outcome	Effect size	95% CI/p-value	Source
Domain-specific					
Global cognition	Social isolation	Cognitive function	r = 0.054	0.043–0.065	Evans et al., 2018 (4)
Global cognition	Loneliness	Cognitive function	r = -0.10	-0.13 to -0.06	Lee et al., 2025 (25)
Episodic memory	Loneliness	Memory decline	r = -0.073	-0.108 to -0.038	Lee et al., 2025 (25)
Executive function	Loneliness	Executive decline	r = -0.108	-0.151 to -0.064	Lee et al., 2025 (25)
Processing speed	Loneliness	Processing speed decline	r = -0.108	-0.151 to -0.064	Lee et al., 2025 (25)
Informant-rated decline	Loneliness	Cognitive decline	r = 0.16	0.14- 0.17	Lee et al., 2025 (25)
Population subgroups					
Women (China)	Social isolation	Episodic memory	β = -0.08	p < 0.001	Guo et al., 2021 (36)
Men (China)	Social isolation	Episodic memory	β = -0.03	Non-significant	Guo et al., 2021 (36)
Rural (24 countries)	Social isolation	Global cognition	β = -0.07	-0.08 to -0.05	Zhang, Zhang, & Gao, 2025 (1)
Urban (24 countries)	Social isolation	Global cognition	β = -0.05	-0.07 to -0.03	Zhang, Zhang, & Gao, 2025 (1)
SCD-MCI	Loneliness	Annual cognitive decline	Cohen’s d = -0.801	p = 0.05	Zhang et al., 2022 (28)
Alzheimer’s disease	Loneliness	Annual cognitive decline	Non-significant	—	Zhang et al., 2022 (28)
Dementia risk					
All-cause dementia	Loneliness	Incident dementia	HR = 1.31	1.19–1.44	Luchetti et al., 2024 (15)
Alzheimer’s disease	Loneliness	Incident AD	HR = 1.39	1.14–1.68	Luchetti et al., 2024 (15)
Vascular dementia	Loneliness	Incident VaD	HR = 1.74	1.11–2.71	Luchetti et al., 2024 (15)
CIND/CI	Loneliness	Incident impairment	HR = 1.15	1.05–1.27	Luchetti et al., 2024 (15)
Intervention effect sizes					
CBT for loneliness	Loneliness intervention	Loneliness reduction	d = -0.598	—	Masi et al., 2011 (5)
RCT loneliness interventions	Loneliness intervention	Loneliness reduction	d = -0.198	—	Masi et al., 2011 (5)
Digital CBT (dCBT)	dCBT intervention	Loneliness reduction	SMD = -0.27	-0.42 to -0.12	Fu et al., 2026 (37)
Non-technology vs. technology	Intervention comparison	Loneliness reduction	Δd = -0.35	p = 0.029	Duffner et al., 2024 (26)
Reminiscence therapy	Loneliness intervention	Loneliness reduction	g = -2.22	—	Lin et al., 2024 (38)
Reminiscence therapy	Loneliness intervention	Cognition improvement	g = 0.34	—	Lin et al., 2024 (38)

AD, Alzheimer’s disease; VaD, vascular dementia; CIND, cognitive impairment no dementia; CBT, cognitive-behavioural therapy; RCT, randomized controlled trial; dCBT, digital cognitive-behavioural therapy; SMD, standardized mean difference; SCD, subjective cognitive decline; MCI, mild cognitive impairment; HR, hazard ratio; 95% CI, 95% confidence interval.

### 3.1 Domain-specific heterogeneity

Across studies, associations are generally small and differ by cognitive domain. Individual-participant meta-analysis linked social connection measures with slower decline in memory, language, and executive function, but the specific social exposure associated with each domain varied (17). Other pooled estimates associated lower isolation with better global cognition, memory, and executive function, while loneliness showed its largest associations with executive function and processing speed (4, 25). These differences may reflect genuine domain sensitivity, but they may also arise from unequal outcome batteries, follow-up intervals, and analytic adjustment.

Conflicting findings are especially evident for memory and temporal direction. Some studies associate loneliness with faster episodic-memory or visuospatial decline, while others report null associations (39). Bidirectional models suggest that memory and loneliness may influence each other across time (40). Within-person analyses have sometimes isolated a prospective association for social isolation after stable individual differences were controlled (41). Together, these studies favour repeated exposure and outcome measurement, but they do not eliminate reverse causation or residual confounding.

### 3.2 Heterogeneity across population subgroups

Age and cognitive stage appear to modify associations, although the direction is not uniform. Social isolation was not associated with incident dementia among adults aged 85 years or older after competing mortality was considered (18), whereas a 24-country analysis reported stronger cognitive associations after age 75 (1). Differences in survival, baseline cognition, follow-up, and exposure measurement may account for part of this inconsistency.

Sex-stratified findings suggest a possible distinction between transitions and persistence rather than a simple male-female difference. In one Chinese cohort, isolation predicted four-year episodic-memory decline only among women (36). Another study associated new-onset loneliness with dementia risk in men and persistent loneliness with risk in women (42). These patterns require replication because subgroup analyses may be sensitive to exposure definitions, event counts, and residual confounding.

Place-based findings are similarly context-dependent. Rural residence was associated with stronger isolation-cognition estimates in one multinational analysis (1), and education explained a substantial share of an urban-rural cognitive difference among Chinese empty-nest older adults (43). However, standard indices may undercount informal rural contacts such as neighbour visits and market gatherings. Apparent urban-rural differences may therefore reflect both social structure and measurement.

Comorbidity can amplify vulnerability but also complicate causal interpretation. Cardiovascular disease (CVD) and hearing impairment have been associated with loneliness or poorer cognitive outcomes (2, 13, 44). Hearing impairment may reduce communication and social participation, while prodromal cognitive decline may itself alter both hearing-related function and social behaviour.

Cultural and racial comparisons indicate that the meaning and consequences of social contact may differ across settings. Country-specific loneliness-cognition estimates varied in a harmonized analysis (25), and social technology use buffered isolation-cognition associations among Black but not White older adults in one study (45). These findings support culturally specific hypotheses, but sparse replication and different technology measures limit firm subgroup recommendations (46).

### 3.3 The Chinese context: distinctive patterns

Chinese studies highlight two recurring themes: the possible protective role of intergenerational co-residence and a digital capability gap despite widespread device access. These themes inform the culturally adapted hypotheses in Section 6, but their strength and generalizability remain uncertain.

Data from the China Health and Retirement Longitudinal Study (CHARLS) indicate higher cognitive-impairment prevalence among rural than urban empty-nest older adults, with education explaining 44.09% of the difference (43). Findings from the Chinese Longitudinal Healthy Longevity Survey (CLHLS) suggest that intergenerational co-residence may protect against loneliness (47, 48). Another Chinese study found that social isolation remained associated with memory decline after loneliness was considered (49). Digital exclusion among the oldest adults often reflects limited skills and usability as well as access (9, 50, 51).

### 3.4 Heterogeneity across cognitive stages

Associations may also differ by cognitive stage, but stage-specific evidence is limited. In participants with subjective cognitive decline (SCD) or mild cognitive impairment, loneliness was associated with regional grey-matter measures and faster annual cognitive decline, whereas corresponding associations were not detected in Alzheimer’s disease (28). A meta-analysis associated loneliness with MCI, with larger estimates for new-onset cases (16). These patterns may indicate an earlier intervention window, but they may also reflect stage-dependent reporting, survival, or reverse causation.

### 3.5 The COVID-19 pandemic and dynamic effects

The COVID-19 pandemic created a natural experiment in abrupt social disruption, but it did not remove confounding or reverse causation. A prospective memory-clinic study also documented newly occurring loneliness during the COVID-19 period (52). A 10-year longitudinal study of Chinese older adults reported associations between changes in loneliness and social isolation and cognitive-functional impairment (53). Six of seven longitudinal studies in one review associated pandemic-related loneliness or isolation with poorer cognition (54). Other longitudinal studies associated new-onset, transient, and persistent disconnection with subsequent decline (14). Entry into isolation showed a larger adverse association than the benefit associated with leaving it in one study (55). These dynamic results strengthen temporal plausibility, yet causal interpretation still depends on exposure measurement, baseline cognition, and adjustment for concurrent health changes.

## 4 Sources of heterogeneity: moderators and mechanisms

### 4.1 Mechanistic divergence between social isolation and loneliness

Social isolation has been associated with reduced cognitive stimulation and lower cognitive reserve, while loneliness has been associated with affective and stress-related processes (5, 19, 28, 29, 32). Longitudinal imaging studies linked greater isolation with smaller hippocampal volumes, but these associations do not establish that isolation caused the structural differences (19, 32). Prodromal cognitive decline may reduce participation and contribute to both isolation and brain change. Intervention evidence more directly supports cognitive-behavioural approaches for reducing loneliness, whereas evidence that increasing contact prevents cognitive decline remains limited (5). The proposed pathways and sources of heterogeneity are summarized in **Figure 1**.

**Figure 1:**
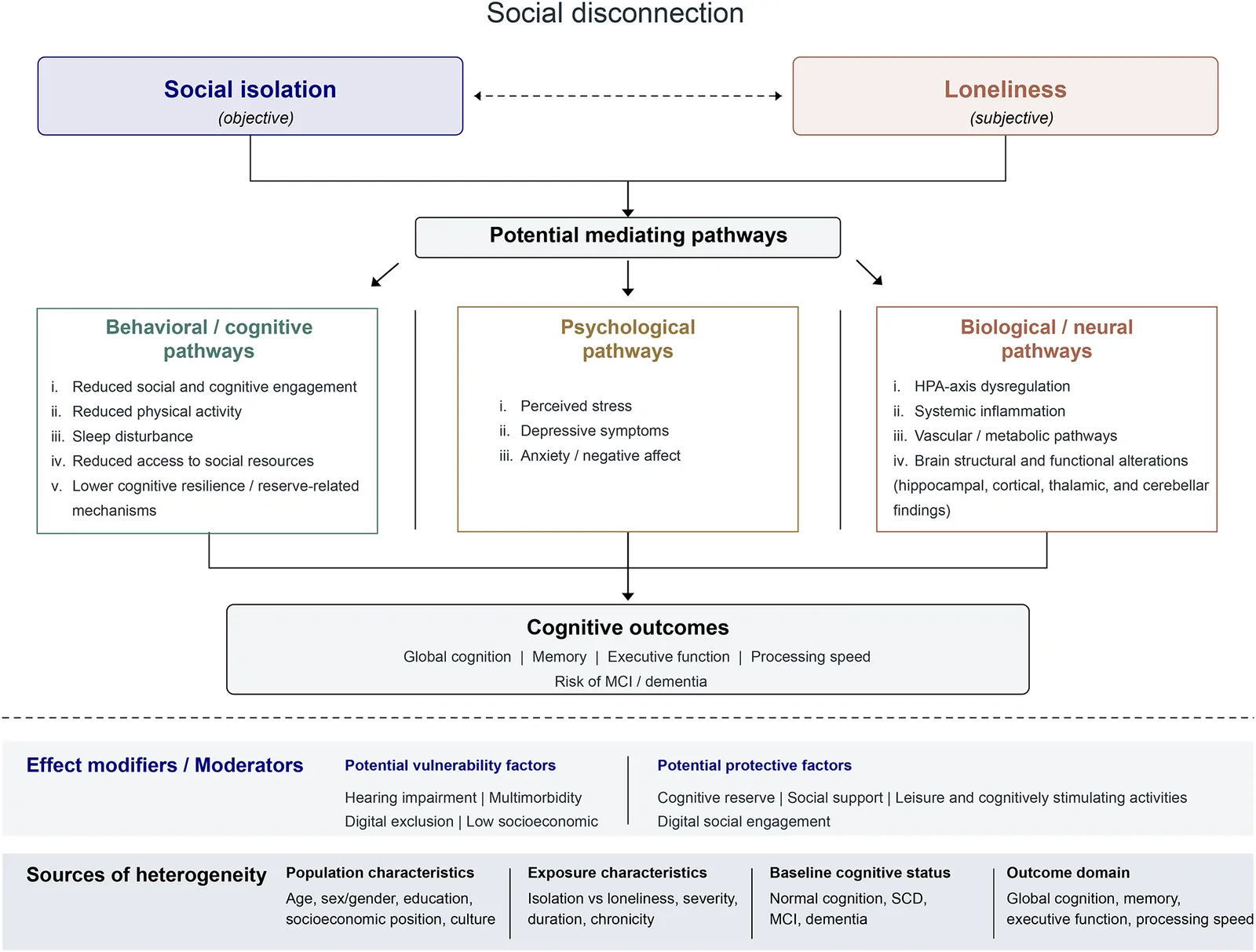
Conceptual framework for the heterogeneous associations linking social isolation, loneliness, and cognitive ageing in older adults. Arrows denote proposed pathways rather than validated causal effects.

### 4.2 Negative moderators: amplifying factors

Hearing loss. Difficulty hearing in background noise was associated with 29% increased odds of loneliness, and hearing aid use was associated with reduced loneliness (2, 56–58). Lampraki et al. (44) found the strongest hearing-cognition associations among those who were non-isolated but lonely.

Digital exclusion has been associated with depression and cognitive outcomes, although definitions vary across studies (50). In one longitudinal cohort of 8,189 older adults, digital isolation was associated with a 36% higher dementia risk (hazard ratio 1.36, 95% confidence interval 1.16 to 1.59) (59). Because this estimate comes from a single observational cohort, it should be interpreted in the context of residual socioeconomic, health, and reverse-causation pathways rather than as a causal effect.

Chronic diseases. Among individuals with CVD, loneliness was associated with higher mortality (HR 1.91) (13). Diabetes was also associated with greater odds of both loneliness and inadequate social support (2).

### 4.3 Positive moderators: buffering factors

Cognitive reserve and resilience. Cognitive reserve was associated with a weaker isolation-cognition relationship in one study, with the interaction concentrated among people with lower occupational complexity (4, 60). This finding supports effect modification as a hypothesis but does not establish that late-life social engagement increases reserve.

Leisure activities and social support. Weekly community group engagement predicted slower memory decline (b = 0.030); living with others predicted slower decline in global cognition, memory, and language (17).

Social media use. Li et al. (51) found that internet use buffered the adverse associations of social isolation with both depression and cognitive function, with stronger effects in males (interaction β = 0.17). Chen et al. (61) quantified a weak but significant negative correlation between social media use and loneliness (r = -0.063), substantially stronger in developing regions (r = -0.242) than developed regions (r = -0.033).

## 5 Mediating mechanisms: from psychosocial to neurobiological pathways

This section examines candidate pathways through which social disconnection may be associated with cognition (**Figure 2**). We distinguish pathways supported by formal mediation analyses from those inferred from longitudinal association, neuroimaging correlation, or biological plausibility.

**Figure 2:**
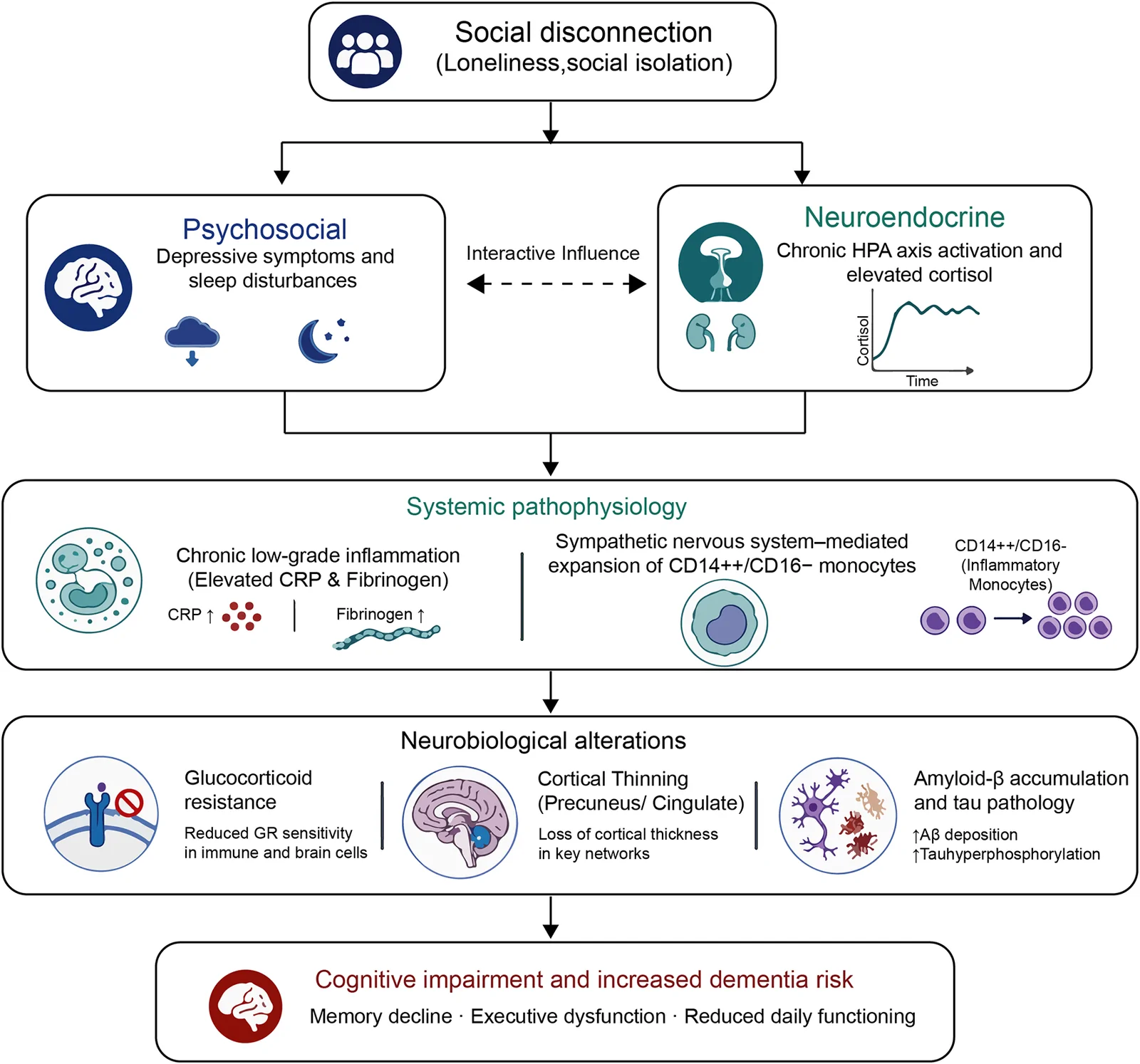
Biological embedding of social disconnection. The diagram summarizes candidate psychosocial, neuroendocrine, inflammatory, and neurobiological pathways rather than validated causal mediation.

### 5.1 Clarifying the role of depression

Depression may act as a mediator, confounder, common cause, or prodromal correlate of both loneliness and cognitive decline (62–64). Formal longitudinal mediation analyses provide some support for indirect pathways, but their causal interpretation depends on temporal ordering and adequate control of time-varying confounding. For example, one Chinese cohort estimated that social isolation mediated 3.1% of the association between depressive symptoms and cognition (65). We therefore treat depression-related mediation as plausible but not established across populations.

### 5.2 Psychosocial and behavioural mediators

Formal mediation evidence is available for only part of the proposed psychosocial pathway. Depressive symptoms accounted for 41% to 79% of the social relationship-dementia association in one prospective analysis (66), and other longitudinal work linked cumulative loneliness, depression, and memory (67). Reduced cognitive stimulation, physical inactivity, and poor sleep are biologically plausible pathways, but most cited studies did not test them simultaneously as mediators.

### 5.3 Neuroendocrine and immune mediators

Loneliness has been associated with hypothalamic-pituitary-adrenal (HPA) axis activation, cortisol, and inflammatory markers (11, 12, 68). In older men, C-reactive protein (CRP) and fibrinogen statistically mediated 6.1% and 12.0%, respectively, of the isolation-cognition association in one study (69). Other work links loneliness with monocyte-related transcriptional patterns (70). These findings support candidate pathways, but they do not establish a universal neuroendocrine mechanism.

### 5.4 Brain structural and functional mediators

Hippocampal volume, cortical thickness, amyloid, tau, and regional grey-matter measures have been associated with social isolation or loneliness (19, 23, 32, 71–73). One analysis estimated that grey-matter volume mediated part of the loneliness-cognition association (74). Most evidence remains observational, and structural or pathological changes may also precede social withdrawal or altered loneliness. Prospective studies with repeated exposure, imaging, and cognitive measurements are needed to test directionality.

### 5.5 Cascading interplay of multiple mediators and the dynamic-acute versus chronic distinction

The reported asymmetry between entering and leaving isolation may reflect different acute and chronic processes, but the underlying biology has not been directly tested (55). It therefore motivates, rather than validates, prevention-focused intervention studies.

Acute isolation could be accompanied by stress-related inflammatory changes, whereas persistent isolation could be associated with longer-term behavioural or neural adaptation (19, 70). These possibilities remain mechanistic hypotheses. No study identified in this review modelled all proposed mediators within one longitudinal framework. Future studies should use repeated measures and formal mediation designs to determine whether different pathways identify clinically useful intervention targets.

## 6 Heterogeneity-based intervention strategies

This section translates the preceding evidence into a research agenda rather than a clinical algorithm. Strategies are grouped by direct randomized controlled trial (RCT) evidence, observational inference, or mechanistic hypothesis. Population strata and feasibility considerations are used to identify questions for prospective validation, not to recommend routine stratified care (**Figure 3**).

**Figure 3:**
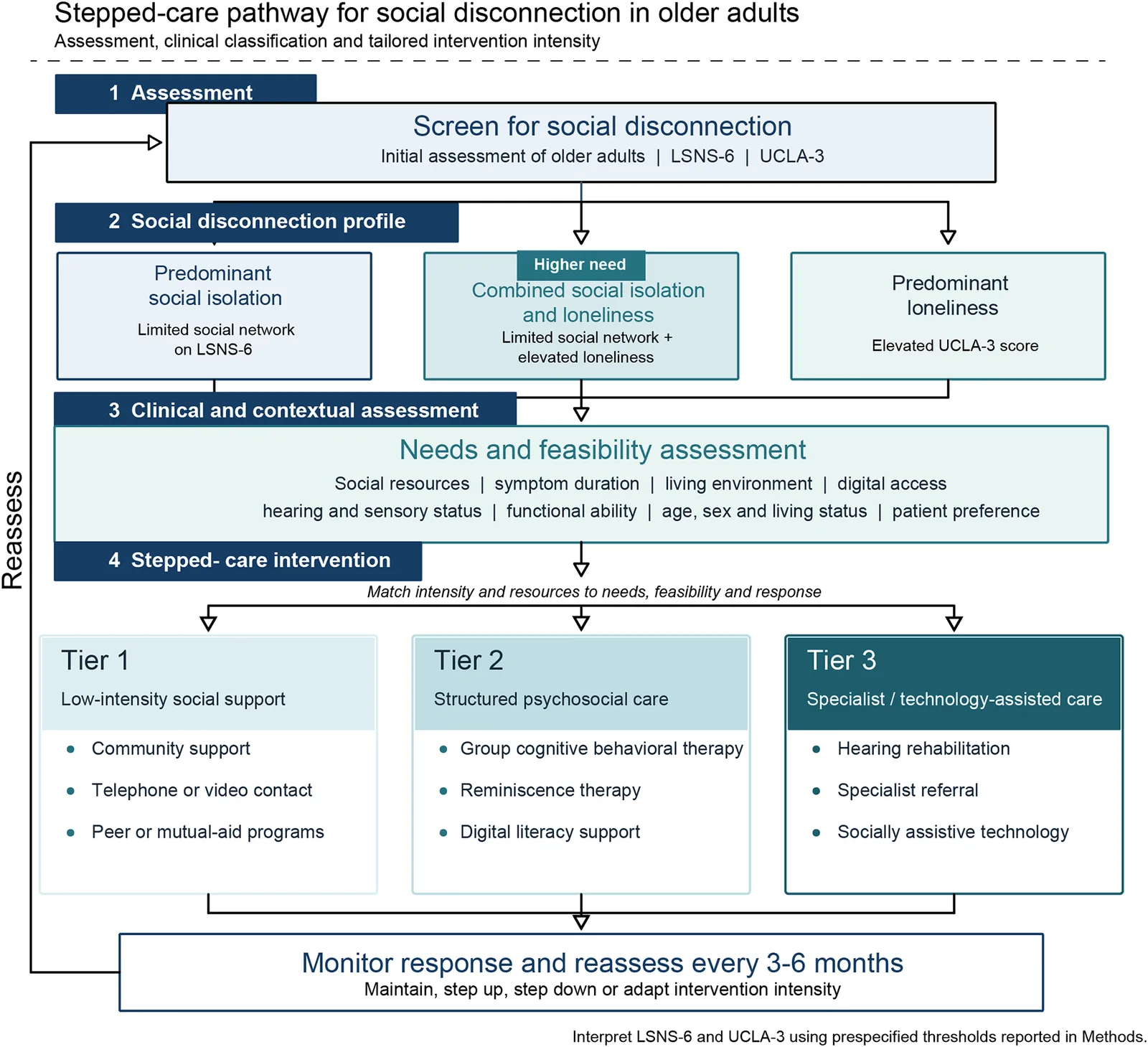
Hypothesis-generating framework for stratified intervention trials addressing social disconnection in older adults. The framework is not a validated clinical triage algorithm.

### 6.1 Evidence level statement and research roadmap

Three evidence levels are distinguished. First, RCT evidence supports some approaches, particularly cognitive-behavioural therapy (CBT), for reducing loneliness (5). Second, observational studies generate hypotheses about urban-rural, sex, and cognitive-stage differences (1, 28, 36, 42). Third, neuroimaging and biological findings suggest possible mechanism-targeted approaches but do not establish intervention efficacy. Only the first category supports current efficacy statements, and even there cognitive or dementia outcomes remain insufficiently tested.

A three-stage research sequence could move these hypotheses toward practice. Stage 1 would replicate moderator effects in independent cohorts. Stage 2 would test prespecified subgroup effects in existing or new RCTs. Stage 3 would evaluate prospectively stratified interventions with clinically relevant cognitive outcomes. Evidence identified in this review does not yet support completion of the third stage.

### 6.2 Precision interventions for diverse populations

Urban-rural differences could inform trial design, but not current treatment allocation. Rural trials could compare interventions that strengthen existing informal networks with programmes imported from urban settings (1). Urban trials could test whether CBT-based approaches are more effective when subjective loneliness is the dominant concern (66).

Sex-specific findings should be treated as hypotheses for prespecified testing. Trials could examine whether new-onset and persistent loneliness require different intervention intensity and whether these effects differ by sex (36, 42, 75). The reported inflammatory pathway in men (69) is not sufficient to recommend anti-inflammatory treatment for loneliness or cognitive prevention.

SCD and MCI may be informative stages for prevention trials because some associations appear stronger before dementia (16, 28, 76). Prospective trials are needed before social interventions can be recommended specifically for these stages.

### 6.3 Mechanism-targeted interventions

For objective isolation, candidate trial components include community participation, walking groups, volunteering, hearing assessment, and digital-literacy support (2, 26, 44, 59, 75, 77). Their effects on cognitive outcomes, rather than only social contact, require direct evaluation.

For loneliness, CBT, cognitive reframing, reminiscence, and digital CBT have reduced loneliness in trials or meta-analyses (5, 37, 38). Reported cognitive benefits are less certain, and effect sizes should not be compared directly across different populations, outcomes, and designs. Social-robot studies remain heterogeneous in setting and intervention content (32).

For depressive symptoms, screening and treatment may be relevant within multicomponent trials because formal mediation analyses link depression with social relationships and dementia risk (65, 66). The optimal sequence and cognitive benefit of such components remain uncertain.

For inflammatory and lifestyle pathways, socially delivered physical activity is a plausible intervention component (2). Anti-inflammatory treatment should not be inferred from observational mediation findings and requires separate clinical evidence (69).

### 6.4 Digital interventions and the digital divide

Digital interventions show small effects on loneliness in some syntheses, including digital CBT and conversational agents (37, 78). Non-technology interventions produced larger pooled effects in one comparison (26), but differences in participants and study design limit direct ranking. The 36% higher dementia-risk estimate for digital isolation came from one observational cohort (59). It supports further research on digital access, skills, and age-friendly interfaces, but not a causal dementia-prevention claim.

### 6.5 Multicomponent integrated interventions

Multicomponent interventions that combine social contact, skills training, and cognitive restructuring have shown benefits for social outcomes (66). The I-CONECT trial reported improved global cognition among socially isolated participants with normal cognition or MCI (76), while a review of artificial-intelligence applications found loneliness reductions in six of nine studies (79). A separate systematic review and meta-analysis also found that artificial-agent interventions may reduce loneliness in older adults (80). These findings justify replication and component testing but do not yet establish an optimal precision-intervention package.

### 6.6 Triage logic for future pilot testing

The following framework is intended only to generate hypotheses for future pilot studies. It has not been validated as a clinical triage algorithm, and its subgroup rules should be tested prospectively before use in practice.

Future studies could evaluate the following stratification hypotheses:

Predominant objective isolation could be used to test community activation and hearing-assessment components.

Predominant subjective loneliness could be used to test CBT or reminiscence components.

New-onset loneliness could be examined as a time-sensitive intervention target, with sex treated as a prespecified effect modifier.

Persistent loneliness could be examined as a marker for longer-duration support, with sex differences tested rather than assumed.

Rural residence and limited digital literacy could define implementation strata for comparing telephone, in-person, and digital approaches.

SCD or MCI could define earlier cognitive stages for trials, without implying proven prevention efficacy.

### 6.7 Implementation feasibility and intervention prioritization

For research planning, interventions can be grouped by relative implementation complexity and evidence maturity. These categories are not validated cost-effectiveness rankings and should not be used as scaling recommendations without prospective evaluation.

Lower-complexity candidates include community activities, telephone check-ins, mutual-aid groups, and video contact (81). Moderate-complexity candidates include group CBT and digital-literacy training. Higher-complexity candidates include hearing rehabilitation and social robots. Comparative trials should assess effectiveness, acceptability, equity, and cost-effectiveness across settings (**Table 3**).

**Table 3:** Candidate intervention strategies by implementation complexity and evidence level.

Strategy category	Specific interventions	Implementation cost	Evidence level	Suggested research status
Lower-complexity candidates				
Community activity organization	Neighbourhood mutual-aid groups and older-adult association activities	Low	Observational (moderate)	Promising; test prospectively before scaling
Telephone check-ins	Regular volunteer/social worker telephone visits	Low	Observational (moderate)	Promising; test prospectively before scaling
WeChat/video calls	Remote family and social connection	Low (basic equipment needed)	RCT (low-moderate)	Evaluate in context-specific trials
Moderate-complexity candidates				
CBT groups	CBT delivered by trained primary mental health workers	Medium (training required)	RCT (high)	RCT-supported for loneliness; assess cognitive outcomes and stratification
Digital literacy training	Smartphone/computer training for older adults	Medium (equipment required)	Observational (moderate)	Evaluate in prospective trials
Higher-complexity candidates				
Hearing aids/rehabilitation	Hearing screening + hearing aid provision	High (specialists required)	Observational (moderate)	Evaluate integrated screening and rehabilitation pathways
Social robots	Paro, BOCCO emo, and similar devices	High (>30,000 RMB/unit)	RCT (moderate)	Assess effectiveness, acceptability, and cost-effectiveness

CBT, cognitive-behavioural therapy; RCT, randomised controlled trial; RMB, Renminbi.

## 7 Research limitations and future directions

### 7.1 Current research limitations

The evidence base has several limitations. Measurement heterogeneity affects both exposure and outcome estimates (15, 23, 25). Directionality remains uncertain because cognitive decline may precede or follow social disconnection (40, 41, 55). Depression may be a mediator, confounder, or prodromal correlate. Many cohorts use single baseline exposure measures, have incomplete confounder control, or lose vulnerable participants during follow-up. Evidence from low- and middle-income countries remains limited, and publication bias may favour positive associations.

Transferability is also uncertain because most cohorts were conducted in high-income countries. Cultural norms, household structure, education, measurement validity, and health-care access differ across settings. Null and domain-specific findings may be underrepresented in the published literature, and this narrative review did not conduct a quantitative publication-bias analysis. Conclusions should therefore be interpreted as a structured synthesis of heterogeneous evidence rather than universal effect estimates.

### 7.2 Future research directions

A high-priority research need is a prospectively stratified RCT programme with prespecified moderators, including type of social disconnection, cognitive stage, and setting. Prevention before persistent isolation is a testable hypothesis arising from transition studies, not an established clinical benefit (55).

Other priorities include harmonized measurement, larger longitudinal neuroimaging studies with repeated assessments, formal models of multiple mediators, replication of sex-specific inflammatory findings, and clearer characterization of people who are isolated but not lonely (35).

Implementation research should examine cost-effectiveness, equity, and acceptability in resource-limited settings, alongside qualitative work on lived experience and intervention preferences.

China-specific priorities include nationally representative longitudinal research with adequate rural and lower-education representation, validation of culturally adapted measures, and prospective evaluation of intergenerational and community-based approaches. Digital mentoring, time-banking, and integration of social-health assessment into public health services should be tested before large-scale adoption.

## 8 Discussion

### 8.1 Summary of principal findings

This narrative review with systematic search elements identifies substantial heterogeneity in associations between social isolation, loneliness, and cognitive health. The two constructs differ conceptually and may involve different correlates, but evidence for distinct causal pathways remains incomplete. Dynamic studies associate transitions into isolation with worse cognitive outcomes, while the isolated but not lonely subgroup may have elevated risk in some cohorts (35, 55). These findings support targeted research questions rather than definitive treatment rules.

### 8.2 Theoretical implications

The synthesis is consistent with cognitive reserve and socioemotional selectivity theories, but it does not show that late-life social engagement directly increases reserve. The elevated risk reported among people who are isolated but not lonely raises the possibility that structural contact and subjective experience contribute differently (35). Neuroimaging and inflammatory findings support a biopsychosocial research model, while reverse causation and residual confounding remain credible alternatives (11, 19, 28, 70).

### 8.3 Clinical implications

Separate assessment of social isolation and loneliness could be evaluated within geriatric care because the constructs may identify different needs. Brief tools such as the LSNS-6 and the three-item UCLA Loneliness Scale (UCLA-3) may support such assessment. However, evidence is insufficient to recommend a universal triage pathway or to conclude that screening-linked interventions prevent cognitive decline. Sensory impairment and recent changes in social connection are reasonable factors for future implementation studies.

### 8.4 Public health implications

Population strategies should distinguish evidence-supported loneliness interventions from broader proposals that remain untested for cognitive outcomes. Community, telephone, and digital approaches may differ in effectiveness, access, and acceptability across settings (26, 50). The observational association between digital isolation and dementia supports further study, not a direct causal prevention claim (59). Policies should therefore pair access initiatives with prospective evaluation of digital skills, usability, equity, and cognitive outcomes. A longitudinal study of Korean older adults examined associations between social connectedness and cognitive function before and during COVID-19 (82).

### 8.5 Limitations of this review

Several limitations also relate specifically to the design of this review. Although a structured search strategy was used, the selection, weighting, and interpretation of evidence in a narrative review inevitably involve reviewer judgement and may introduce selection and interpretive bias. The search was restricted to English- and Chinese-language literature, which may have resulted in language bias. In addition, the substantial heterogeneity in exposure definitions, measurement instruments, and cognitive outcomes limited direct comparison across studies. Because this was not a quantitative meta-analysis, we did not generate a pooled estimate or formally assess publication bias using statistical methods such as funnel plots or Egger-type tests.

### 8.6 Causal interpretation and residual uncertainty

Most included evidence is observational. Temporal ordering, residual socioeconomic and health confounding, selective attrition, and reverse causation remain plausible across the proposed pathways. Accordingly, the review interprets reported estimates as associations unless supported by randomized or formal longitudinal mediation evidence. The precision framework should be judged as a programme for prospective testing rather than a validated prevention model.

## 9 Conclusion

This review synthesizes heterogeneous associations between social isolation, loneliness, and cognitive health. The distinction between objective isolation and subjective loneliness is conceptually well supported, while evidence for separate neurobiological pathways remains preliminary. Associations vary across cognitive domains, populations, stages, and measurement approaches. Dynamic studies improve temporal understanding but do not eliminate reverse causation or residual confounding.

The evidence supports stratified research rather than immediate precision prescribing. Intervention trials have established benefits for some loneliness outcomes, but effects on cognitive decline and dementia remain uncertain. Population, mechanism, and delivery-platform hypotheses should therefore be tested in prespecified prospective trials.

Clinical implications. Separate assessment of social isolation and loneliness may help identify different support needs, but screening-linked cognitive benefits have not been established. Research should test whether recent changes in social connection identify modifiable risk before persistent isolation develops.

Public health implications. Age-friendly communities, intergenerational connection, and digital inclusion are plausible components of broader healthy-ageing policy. Their effectiveness for dementia prevention should be evaluated directly, with attention to non-digital alternatives, equity, acceptability, and context.

The central challenge is to convert heterogeneous observational evidence into scalable interventions without overstating causality. Progress will require harmonized measures, transparent synthesis, longitudinal and randomized designs, and culturally grounded implementation research.
